# DDX4 enhances antiviral activity of type I interferon by disrupting interaction of USP7/SOCS1 and promoting degradation of SOCS1

**DOI:** 10.1128/mbio.03213-23

**Published:** 2024-02-20

**Authors:** Ying Miao, Tingting Zhang, Mingcheng Guan, Qian Zhao, Renxia Zhang, Xuyi Liu, Tianrun Ma, Tengfei Ren, Zhijin Zheng, Wei He, Wanying Tian, Qun Cui, Xingyu Zhai, Yibo Zuo, Hong Zhu, Hui Zheng, Yukang Yuan

**Affiliations:** 1 Jiangsu Key Laboratory of Infection and Immunity, Institutes of Biology and Medical Sciences, Soochow University, Suzhou, Jiangsu, China; 2 International Institute of Infection and Immunity, Institutes of Biology and Medical Sciences, Soochow University, Suzhou, Jiangsu, China; 3 Department of Medical Oncology, The First Affiliated Hospital of Soochow University, Suzhou Medical College of Soochow University, Suzhou, China; 4 Department/Institute of Laboratory Medicine, Sichuan Provincial People’s Hospital, Medical School, University of Electronic Science and Technology of China, Chengdu, Sichuan, China; Icahn School of Medicine at Mount Sinai, New York, USA

**Keywords:** antiviral activity, interferon, DEAD-box helicase 4, suppressor of cytokine signaling 1, ubiquitin-specific protease 7

## Abstract

DEAD-box helicase (DDX) family members play differential roles in regulating innate antiviral immune response. However, the physiological roles played by DDX4 in antiviral innate immunity remain unclear. In this study, we unveiled that DDX4 acts as a positive regulatory molecule of Type-I interferon (IFN-I)-mediated antiviral activity. Our findings demonstrate that IFN-I upregulates DDX4 protein levels, and subsequently, overexpression of DDX4 enhances the IFN-I-mediated signaling pathway. This creates a positive feedback loop that amplifies the antiviral response. DDX4 was found to bind with deubiquitinase ubiquitin-specific protease 7 (USP7), leading to the disruption of the interaction between USP7 and suppressor of cytokine signaling 1 (SOCS1) and the subsequent degradation of SOCS1. This process enhances the antiviral function of IFN-I. Our findings provide new insights into the regulatory role of DDX4 in the IFN-I response.

**IMPORTANCE:**

DDX4, identified as a putative RNA helicase that modulates RNA secondary structure through RNA binding, is primarily acknowledged for its role in regulating mRNA translation within the germline. Nevertheless, the extent of DDX4’s involvement in the antiviral innate immune response remains largely unexplored. This study presents evidence of a previously unrecognized positive feedback loop between DDX4 and the antiviral response, suggesting that disruption of this loop may serve as a novel mechanism for viral evasion. Furthermore, our findings elucidate a positive regulatory mechanism by which the DDX4/USP7/SOCS1 axis mediates the antiviral activity of Type-I interferon, which provides new insight into strategies for improving the efficacy of IFN-based antiviral therapy.

## INTRODUCTION

Type-I interferon (IFN-I), including IFN-α and IFN-β, serves as a crucial player in mediating the innate immune response against viruses; thus, IFN-I is utilized for the treatment of viral infections in clinics (1). Typically, the interaction between IFN-I and its receptors (IFNAR1 and IFNAR2) expeditiously triggers the Janus kinase (JAK)/signal transducers and activators of transcription (STAT) pathway, resulting in the phosphorylation of STAT1 and STAT2. Following this, the active transcription factor 3 (ISGF3), which comprises STAT1, STAT2, and interferon regulatory factor 9 (IRF9), is formed and subsequently translocated into the nucleus. It then binds to the IFN-stimulated response elements situated in the promoter region of interferon-stimulated genes (ISGs), thereby leading to the transcription and expression of numerous antiviral ISGs.

To date, it has been observed that the signaling pathways mediated by IFN-I are subject to modulation by an increasing array of accessory proteins and posttranslational modifications. Among these modifications, the ubiquitin (Ub)-proteasome system plays a crucial role in regulating the degradation of numerous cellular proteins. Particularly noteworthy are the deubiquitinases, which possess the ability to reverse Ub modifications, thereby serving as indispensable regulators of this system. Our previous study has shown that deubiquitinase ATXN3 promotes the IFN-I antiviral response by deubiquitinating histone deacetylase 3 (2). The antiviral activity of IFN-I also can be positively regulated by the ability of deubiquitinase USP2a to restrict the ubiquitination of activated STAT1 (3). Furthermore, we observed that deubiquitinase USP7 protein negatively regulates IFN-I-mediated antiviral activity by stabilizing suppressor of cytokine signaling 1 (SOCS1) (4). As well, SOCS1 is a negative regulator of IFN, by modulating JAK/STAT pathways or functioning through IFNAR1/2 (5, 6). It follows that Ub-proteasome system plays a significant role in the antiviral defense of IFN-I.

In addition to the Ub-proteasome system, growing data indicate that DEAD-box helicases (DDXs) are implicated in innate immunity through sensing viral nucleic acids or affecting downstream signaling pathways (7, 8). Briefly, DDXs, with more than 12 conserved motifs, are presumed RNA helicases that exert their function by binding to RNA and inducing alterations in RNA secondary structure (9). Notably, our observations indicate a significant upregulation in mRNA levels of only two members of the DDX family, namely DDX4 and DDX58, following IFN stimulation. DDX58, also referred to as RNA sensor RIG-I, has been extensively documented as being implicated in the recognition of viral double-stranded RNA and the regulation of the antiviral innate immune response (10). DDX4, known as VASA, possesses a sequence similar to the eukaryotic initiation factor 4A and is believed to exert a role in governing mRNA translation in the germline (11). Furthermore, DDX4 exhibits expression not only in the germline but also in stem cells and tumor cells (12). Nevertheless, the precise physiological significance of DDX4 in the antiviral innate immune response remains inadequately elucidated.

In this study, we revealed that IFN-I can upregulate the expression of DDX4; in return, DDX4 enhances the IFN-I-mediated antiviral activity by the regulation of downstream signaling pathways. Mechanically, we demonstrated that DDX4 negatively regulates the expression and stability of SOCS1 protein in a USP7-dependent manner by inhibiting the interaction of USP7 with SOCS1, thus strengthening the antiviral function of IFN-I. Our findings might provide new insight into strategies for improving the efficacy of IFN-I-based antiviral therapy.

## RESULTS

### The expression of DDX4 increased by IFN

**Fig 1:**
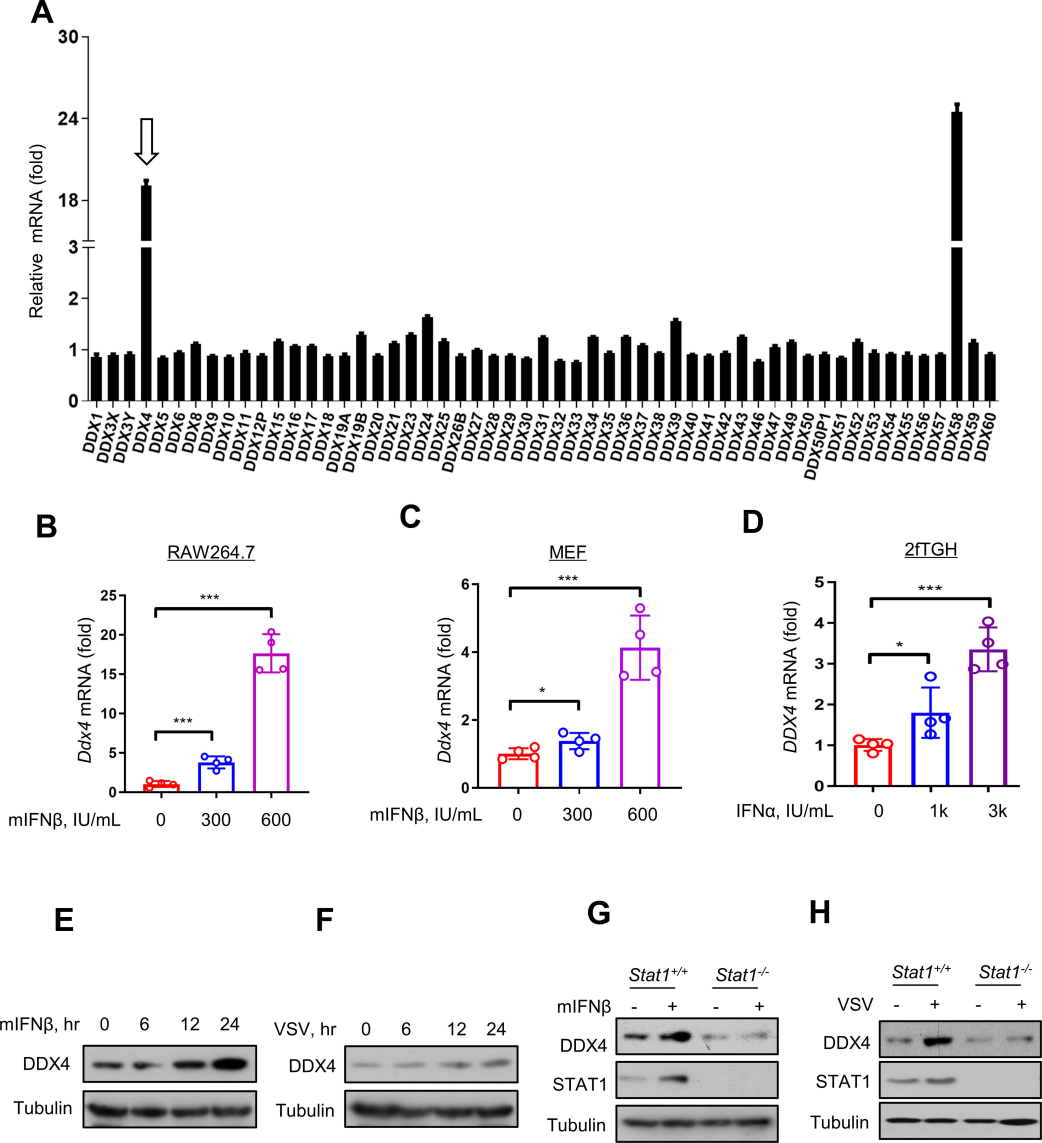
IFN upregulates the expression of DDX4. (**A**) Relative mRNA levels of DDX family proteins stimulated by IFN using the Interferome database (http://www.interferome.org/). (**B and C**) RT-qPCR analysis of *Ddx4* mRNA levels in RAW264.7 (**B**) or MEF (**C**) cells treated with mIFNβ (300 or 600 IU/mL). (**D**) RT-qPCR analysis of *DDX4* mRNA levels in 2fTGH cells treated with IFNα (1,000 or 3,000 IU/mL). (**E**) Western blot analysis of DDX4 protein in RAW264.7 cells treated with mIFNβ (300 IU/mL) for the indicated times. (**F**) Western blot analysis of DDX4 protein in RAW264.7 cells infected with VSV (MOI (Multiplicity Of Infection) = 1.0) for the indicated times. (**G and H**) Western blot analysis of DDX4 and STAT1 proteins in *Stat1^+/+^* or *Stat1^-/-^* RAW264.7 cells treated with mIFNβ (300 IU/mL) (**G**) or infected with VSV (MOI = 1.0) (**H**). Data are shown as mean  ±  SD of four biological replicates (**B–D**) or are representative of three independent experiments (**E–H**). *P < 0.05 and ***P < 0.001.

To explore the impact of IFN on DDX family members, we analyzed mRNA levels of DDX family members stimulated by IFN using the Interferome database (http://www.interferome.org/) (Fig. 1A) (13). In contrast, only two members’ mRNA levels, i.e., DDX4 and DDX58, were significantly upregulated. Reportedly, DDX58 (also known as RIG-I) is an essential pattern-recognition receptor for viral RNA, which is involved in antiviral immunity (14). The role of DDX4 on antiviral activity, however, remained unknown. As such, we measured DDX4 mRNA levels by real-time quantitative PCR (RT-qPCR) analysis in RAW264.7 cells (Fig. 1B) or MEF (Mouse Embryo Fibroblast) cells (Fig. 1C) treated with mouse IFNβ (mIFNβ; 300 and 600 IU/mL), while in 2fTGH cells treated with IFNα (1,000 and 3,000 IU/mL) (Fig. 1D). We observed a significant increase of DDX4 mRNA levels with the rise of the IFN dose. In addition, the temporal effect of IFN on DDX4 protein expression was investigated by western blot analyses. The results showed that the DDX4 protein levels were increased over time in RAW264.7 cells after IFN or Vesicular Stomatitis Virus (VSV) stimulation (Fig. 1E and F). Given the upregulated DDX4 protein by IFN, we further determined whether DDX4 protein serves as a novel ISG. Knockout of *Stat1* blocked DDX4 protein induction by either mIFNβ (Fig. 1G) or viruses (Fig. 1H), indicating that DDX4 is an ISG. In short, IFN upregulates the expression of DDX4.

### The antiviral activity mediated by DDX4

**Fig 2:**
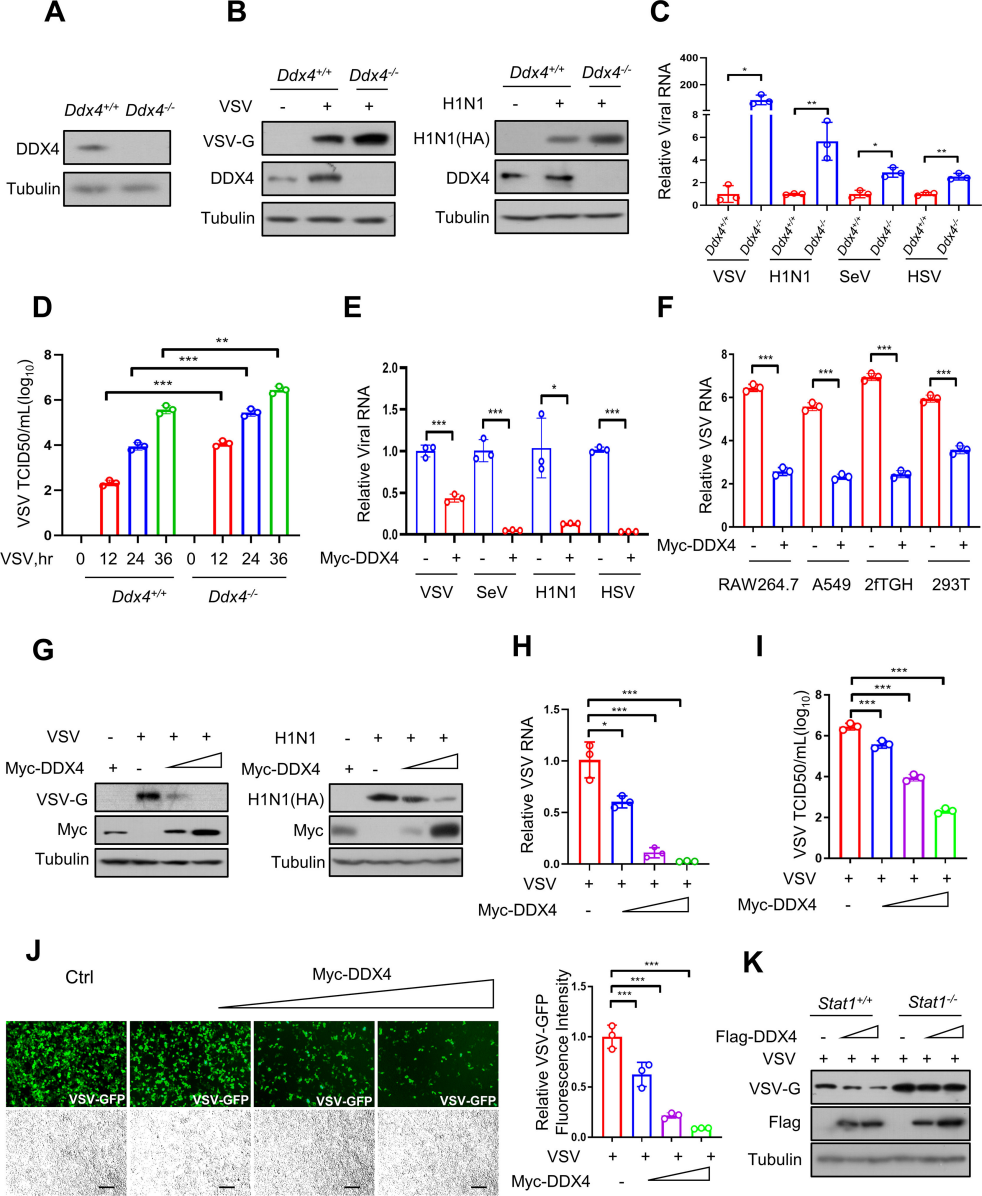
DDX4 mediates antiviral activity. (**A**) Western blot analysis of DDX4 protein in *Ddx4*^+/+^ or *Ddx4*^-/-^ RAW264.7 cells. (**B**) Western blot analysis of VSV-G protein and H1N1-encoded HA protein levels in *Ddx4*^+/+^ or *Ddx4*^-/-^ RAW264.7 cells infected with VSV (left) or H1N1 (right). (**C**) RT-qPCR analysis of viral RNA levels in *Ddx4*^+/+^ or *Ddx4*^-/-^ RAW264.7 cells infected with VSV (MOI = 1.0), H1N1 (MOI = 1.0), SeV (MOI = 1.0), or HSV (MOI = 1.0) for 20 h. (**D**) Viral growth assay of VSV titers in culture supernatants of *Ddx4*^+/+^ or *Ddx4*^-/-^ cells infected with VSV (MOI = 1.0) for 12, 24, or 36 h. (**E**) RT-qPCR analysis of viral RNA levels in HEK293T cells transfected with or without Myc-DDX4 and then infected with VSV (MOI = 1.0), SeV (MOI = 1.0), H1N1 (MOI = 1.0), or HSV (MOI = 1.0) for 20 h. (**F**) RT-qPCR analysis of VSV RNA levels in RAW264.7, A549, 2fTGH, or HEK293T cells transfected with or without Myc-DDX4 and then infected with VSV (MOI = 1.0) for 20 h. (**G**) Western blot analysis of VSV-G protein and H1N1-encoded HA protein levels in HEK293T cells transfected with increasing amounts of Myc-DDX4 and then infected with VSV (left) or H1N1 (right). (**H**) RT-qPCR analysis of VSV RNA levels in HEK293T cells transfected with increasing amounts of Myc-DDX4 and then infected with VSV (MOI = 1.0) for 20 h. (**I**) 50% tissue culture-infective dose assay of VSV titers in culture supernatants of HEK293T cells transfected with increasing amounts of Myc-DDX4 and then infected with VSV (MOI = 1.0) for 20 h. (**J**) Fluorescence microscopy of VSV-GFP viruses and VSV-GFP fluorescence intensity in HEK293T cells transfected with increasing amounts of Myc-DDX4 and then infected with VSV-GFP (MOI = 1.0) 20 h. Scale bars, 100 µm. (**K**) Western blot analysis of VSV-G protein in *Stat1^+/+^* and *Stat1^-/-^* RAW264.7 cells transfected with increasing amounts of Myc-DDX4 and then infected with VSV (MOI = 1.0). Data are shown as mean  ±  SD of three biological replicates (**C, D, E, F, and H**) or are representative of three independent experiments (**A, B, G, and K**). *P < 0.05, **P < 0.01, and ***P < 0.001.

Both IFN and virus can upregulate DDX4, and we next sought to investigate the role of DDX4 in the innate antiviral response. To explore the issue, we first constructed DDX4-knockout stable cell lines (Fig. 2A). Generally, the RNA viruses of VSV, Sendai Virus (SeV), Influenza A virus (H1N1), and the DNA virus of herpes simplex virus (HSV) have been widely used as sensitive viral models to evaluate cellular antiviral activity. After infecting the *Ddx4*^+/+^ or *Ddx4*^-/-^ RAW264.7 cells with VSV (left) or H1N1 (right) viruses, the *Ddx4*^-/-^ cells had higher levels of viral proteins than the wild type cells (Fig. 2B). For further verification, *Ddx4*^+/+^ or *Ddx4*^-/-^ RAW264.7 cells were infected with VSV, H1N1, SeV, or HSV, and the levels of specific viral RNAs were observed by RT-qPCR analysis; the data showed that the viral RNA levels were higher in *Ddx4*^-/-^ cells than in *Ddx4*^+/+^ cells (Fig. 2C). Besides, we infected the cells with VSV for 12, 24, and 36 h, and the viral growth kinetics was quantified; the results showed that *Ddx4*^-/-^ exhibited faster growth kinetics than *Ddx4*^+/+^ cells (Fig. 2D). At the same time, the experiment of overexpressing Myc-DDX4 in cells, and then infected cells with VSV, SeV, H1N1, or HSV also verified that the conclusion of DDX4 promotes a cellular antiviral response (Fig. 2E). Simultaneously, we found that VSV mRNA levels were decreased when overexpressing DDX4 in multiple cells lines including RAW264.7, A549, 2fTGH, and HEK293T (Fig. 2F). These data indicate the broad-spectrum antiviral function of DDX4. For further verification, we overexpressed Myc-DDX4 in cells at varying doses, and our findings indicate that the protein levels of VSV and H1N1 (Fig. 2G), the mRNA levels of VSV (Fig. 2H), the titer levels of VSV (Fig. 2I), and the number of cells infected by VSV-GFP (Green Fluorescent Protein) (Fig. 2J) were all diminished in a dose-dependent manner of Myc-DDX4. Briefly, DDX4 enables mediating antiviral activity. Besides, we assessed viral susceptibility in *Stat1^-/-^* cells overexpressing DDX4, and the result showed that overexpressing DDX4 in *Stat1^-/-^* cells cannot significantly downregulated the protein level of VSV-G (Fig. 2K).

### The IFN-I-mediated signaling pathway promoted by DDX4

**Fig 3:**
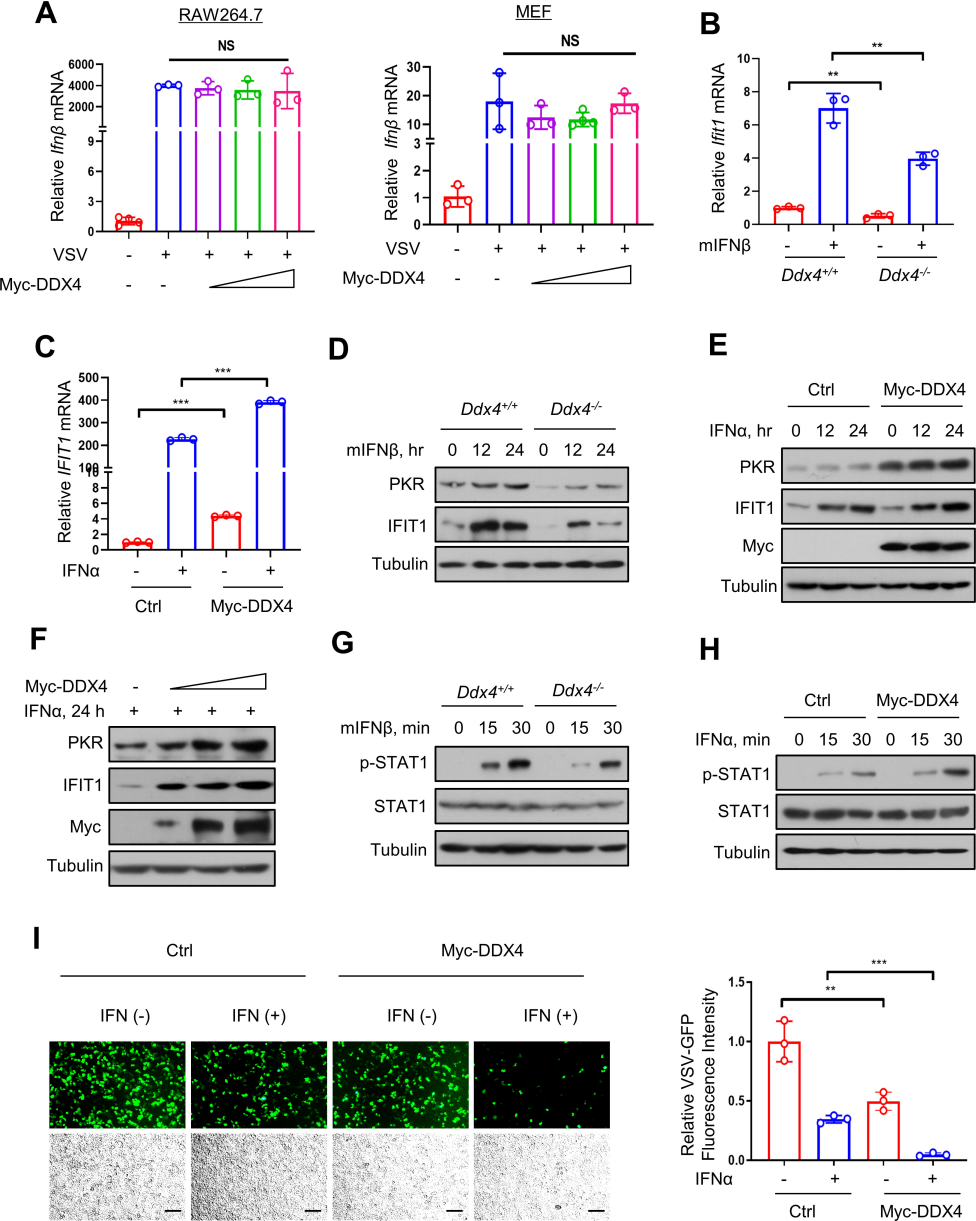
DDX4 enhances the IFN-I mediated signaling pathway. (**A**) RT-qPCR analysis of *Ifnβ* mRNA levels in RAW264.7 (left) and MEF (right) cells transfected with increasing amounts of Myc-DDX4 and then infected with VSV (MOI = 1.0, 10 h). (**B**) RT-qPCR analysis of *Ifit1* mRNA levels in *Ddx4*^+/+^ or *Ddx4*^-/-^ RAW264.7 cells treated with mIFNβ (300 IU/mL) for 8 h. (**C**) RT-qPCR analysis of *IFIT1* mRNA levels in HEK293T cells transfected with empty vectors or Myc-DDX4 and then treated with IFNα (1,000 IU/mL). (**D**) Western blot analysis of PKR and IFIT1 proteins in *Ddx4*^+/+^ or *Ddx4*^-/-^ RAW264.7 cells treated with mIFNβ (300 IU/mL) for the indicated times. (**E**) Western blot analysis of PKR and IFIT1 protein in HEK293T cells transfected with empty vectors or Myc-DDX4 and then treated with IFNα (1,000 IU/mL) for the indicated times. (**F**) Western blot analysis of PKR and IFIT1 protein in HEK293T cells transfected with increasing amounts of Myc-DDX4 and then treated with IFNα (1,000 IU/mL) for 24 h. (**G**) Western blot analysis of p-STAT1 in *Ddx4*^+/+^ or *Ddx4*^-/-^ RAW264.7 cells treated with mIFNβ (300 IU/mL) for the indicated times. (**H**) Western blot analysis of p-STAT1 in HEK293T cells transfected with empty vectors or Myc-DDX4 and then treated with IFNα (1,000 IU/mL) for the indicated times. (**I**) Fluorescence microscopy of VSV-GFP viruses and VSV-GFP fluorescence intensity in HEK293T cells transfected with empty vectors or Myc-DDX4 and then treated with IFNα (50 IU/mL) for 20 h, then infected with VSV-GFP (MOI = 1) for 24 h. Scale bars, 100 µm. Data are shown as mean  ±  SD of three biological replicates (**A, B, and C**) or are representative of three independent experiments (**D–H**). NS, not significant; **P < 0.01 and ***P < 0.001.

Subsequently, we wanted to determine whether DDX4 promotes IFN-I production during viral infection. RAW264.7 and MEF cells were transfected with Myc-DDX4 in a dosed manner and then infected with VSV. The data demonstrated that overexpression of DDX4 did not affect the mRNA levels of IFNβ during viral infection (Fig. 3A). Given the above data, we attempted to probe into the mechanism of DDX4. As such, we tried to analyze whether DDX4 affects the downstream signaling pathway mediated by IFN-I. In general, following the activation of the IFN-I pathway, the transcription factor complex, including p-STAT1, translocates into the nucleus, ultimately inducing the expression of a series of ISGs. We observed that knocking out the endogenous DDX4 significantly blocked the downstream IFN-I activated ISGs, represented by IFIT1 and PKR (Protein Kinase R), both at the mRNA (Fig. 3B) and protein levels (Fig. 3D). In turn, cells were transfected with Myc-DDX4 plasmids, and the overexpression of exogenous DDX4 promoted IFN-mediated IFIT1 mRNA levels (Fig. 3C). This phenomenon was also observed at ISGs protein levels, and the protein levels of PKR and IFIT1 were significantly increased not only at different IFN treatment times (Fig. 3E) but also at different doses of overexpressing Myc-DDX4 (Fig. 3F). Next, we investigated whether DDX4 could regulate STAT1 phosphorylation induced by IFN-I. The data showed that DDX4 deficiency lowered IFN-I-induced STAT1 phosphorylation (Fig. 3G), while overexpression of DDX4 upregulated the phosphorylation level of STAT1 in IFN-I signaling (Fig. 3H). More directly, fluorescence microscopy of VSV-GFP viruses and VSV-GFP fluorescence intensity proved that DDX4 potentiates the antiviral activity of IFN-I (Fig. 3I), indicating that DDX4 plays an important role in promoting the IFN-I-activated signaling pathway. In a word, DDX4 enhances the IFN-mediated signaling pathway.

### The interaction of USP7 with SOCS1 disrupted by DDX4

**Fig 4:**
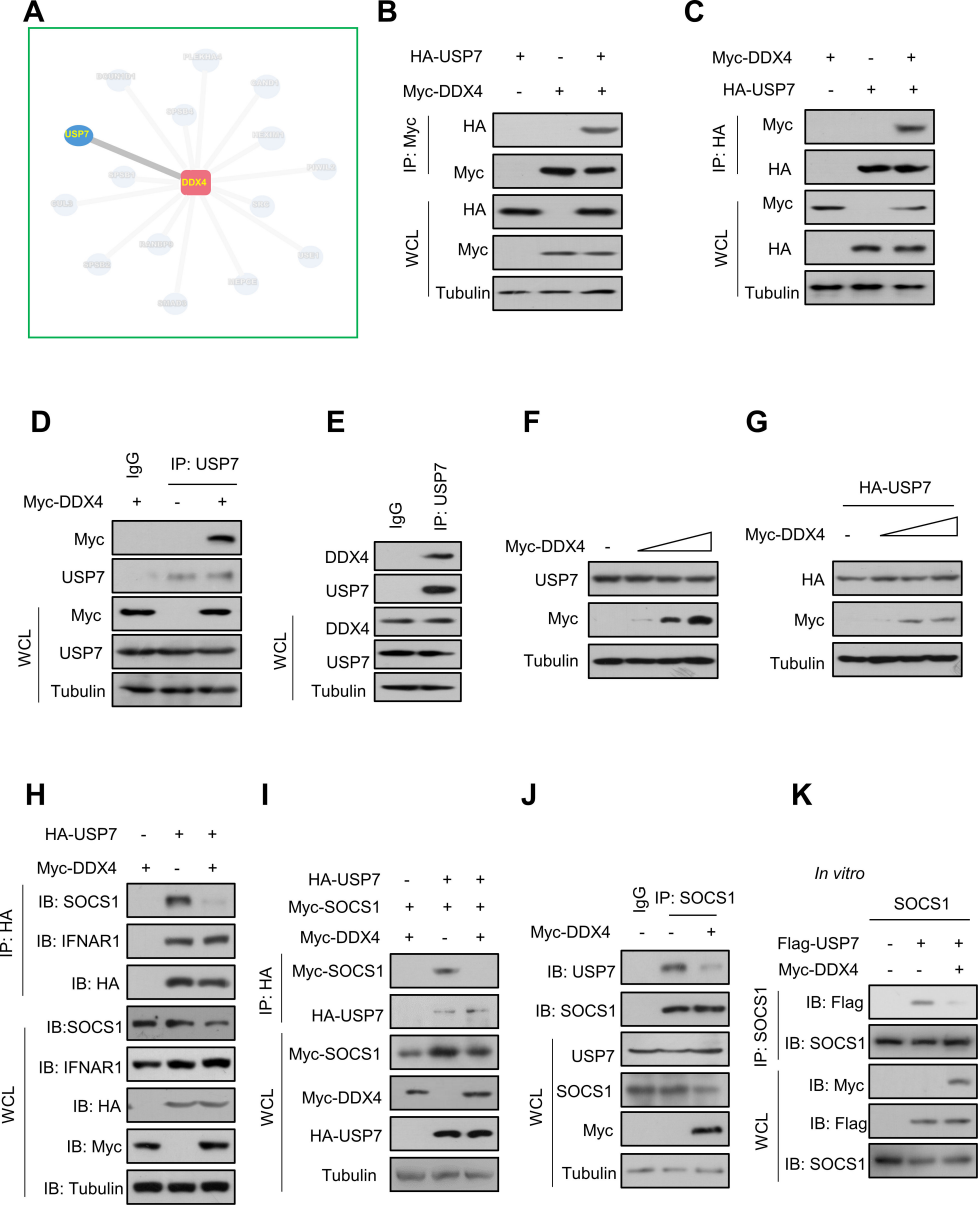
DDX4 inhibits the interaction of USP7 with SOCS1. (**A**) Potential of interaction protein of DDX4 obtained from the PINA database. (**B**) Immunoprecipitation analysis of the interaction between HA-USP7 and Myc-DDX4 by immunoprecipitating Myc-DDX4 in HEK293T cells cotransfected with HA-USP7 and Myc-DDX4. (**C**) Immunoprecipitation analysis of the interaction between HA-USP7 and Myc-DDX4 by immunoprecipitating HA-USP7 in HEK293T cells cotransfected with HA-USP7 and Myc-DDX4. (**D**) Immunoprecipitation analysis of the interaction between endogenous USP7 and Myc-DDX4 in HEK293T cells transfected with Myc-DDX4 using either the isotype IgG (-) or anti-USP7 antibodies (USP7) for immunoprecipitation. (**E**) Immunoprecipitation analysis of the interaction between endogenous USP7 and DDX4 in HEK293T cells using either the isotype IgG (-) or anti-USP7 antibodies (USP7) for immunoprecipitation. (**F**) Western blot analysis of USP7 protein levels in HEK293T cells transfected with an increasing amount of Myc-DDX4. (**G**) Western blot analysis of HA-USP7 levels in HEK293T cells cotransfected with HA-USP7 and an increasing amount of Myc-DDX4. (**H**) Immunoprecipitation analysis of the interaction between endogenous SOCS1, endogenous IFNAR1, and HA-USP7 in HEK293T cells cotransfected with HA-USP7 and Myc-DDX4. (**I**) Immunoprecipitation analysis of the interaction between Myc-SOCS1 and HA-USP7 in HEK293T cells cotransfected with HA-USP7, Myc-SOCS1, and Myc-DDX4. (**J**) Immunoprecipitation analysis of the interaction between endogenous SOCS1 and USP7 in HEK293T cells transfected with or without Myc-DDX4. (**G**) *In vitro* binding assay of the interaction between SOCS1, USP7, and DDX4 by immunoprecipitating SOCS1. Data are shown as mean  ±  SD of three biological replicates (**B–K**).

Given the positive regulation of DDX4 on the IFN-I signaling pathway, we wanted to further determine the potential substrate of DDX4 in regulating IFN-I signaling. As such, we used the PINA2 website to predict the molecules that could interact with DDX4 (Fig. 4A). Interestingly, we noticed a possible interaction between DDX4 and USP7. Our previous study has demonstrated that the USP7 protein interacts with the SOCS1 to increase its stability by deubiquitination effects, thus negatively regulating IFN-I-mediated antiviral activity (4). To validate the website’s predictions, cells were transfected with HA-USP7 and Myc-DDX4. Immunoprecipitation analyses indicated a clear interaction between exogenous HA-USP7 and Myc-DDX4, regardless of the immunoprecipitation of Myc-DDX4 (Fig. 4B) or HA-USP7 (Fig. 4C).

Furthermore, we observed that endogenous USP7 can interact with exogenous DDX4 in cells (Fig. 4D). The interaction between DDX4 and USP7 was also confirmed by dual endogenous experiments (Fig. 4E). However, overexpression of Myc-DDX4 did not affect the protein levels of endogenous USP7 (Fig. 4F) or exogenous USP7 (Fig. 4G). In fact, in addition to SOCS1, USP7 has been reported to interact with IFNAR1 to be involved in regulating IFN-I-mediated antiviral activity (15). As such, we wanted to determine whether DDX4 affects the interaction of USP7 with SOCS1 or not. Immunoprecipitation analysis showed that DDX4 did not inhibit the interaction of USP7 with IFNAR1 but blocked the interaction of USP7 with SOCS1 (Fig. 4H). Further analyses indicated that DDX4 inhibited the interaction of HA-USP7 with Myc-SOCS1 (Fig. 4I). Similarly, we overexpressed Myc-DDX4 in cells, and the interaction between endogenous SOCS1 and endogenous USP7 is significantly reduced (Fig. 4J). Meanwhile, we performed an *in vitro* binding assay, and the result showed that after adding DDX4, the interaction between USP7 and SOCS1 was significantly weakened, which means that DDX4 may outcompete SOCS1 for binding to USP7 (Fig. 4K). Altogether, DDX4 can inhibit the interaction of USP7 with SOCS1.

### SOCS1 ubiquitination levels increased by DDX4 in a USP7-dependent manner

**Fig 5:**
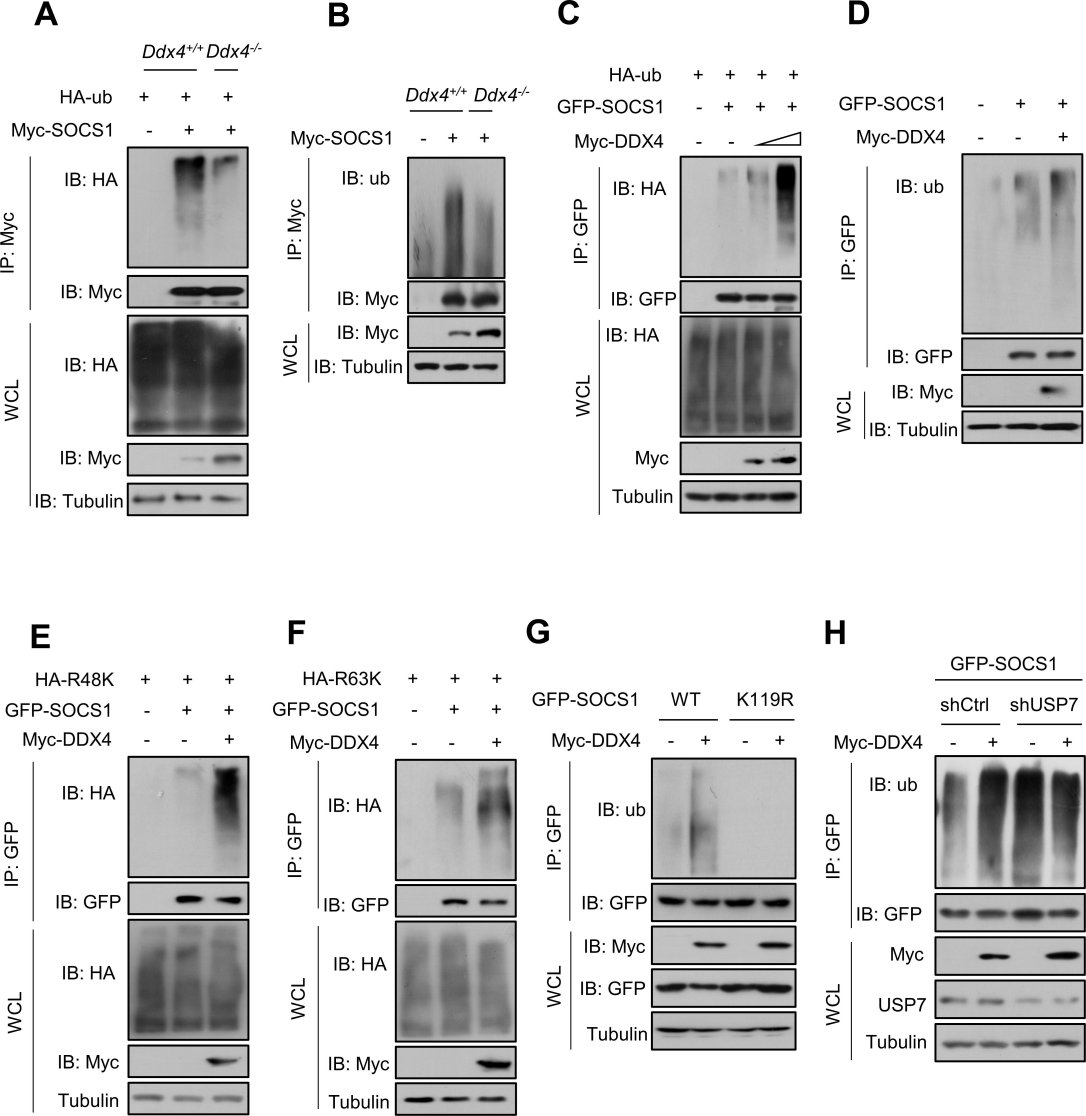
DDX4 regulates SOCS1 ubiquitination levels in a USP7-dependent manner. (**A**) Immunoprecipitation analysis of polyubiquitination of Myc-SOCS1 in *Ddx4*^+/+^ or *Ddx4*^-/-^ RAW264.7 cells cotransfected with Myc-SOCS1 and HA-Ub as indicated and then treated with MG132 (10 µM, 4 h). (**B**) Immunoprecipitation analysis of polyubiquitination of Myc-SOCS1 in *Ddx4*^+/+^ or *Ddx4*^-/-^ RAW264.7 cells transfected with Myc-SOCS1 as indicated and then treated with MG132 (10 µM, 4 h). (**C**) Immunoprecipitation analysis of polyubiquitination of GFP-SOCS1 in HEK293T cells cotransfected with GFP-SOCS1, Myc-DDX4, and HA-Ub as indicated and then treated with MG132 (10 µM, 4 h). (**D**) Immunoprecipitation analysis of polyubiquitination of GFP-SOCS1 in HEK293T cells cotransfected with GFP-SOCS1 and Myc-DDX4 as indicated and then treated with MG132 (10 µM, 4 h). (**E**) Immunoprecipitation analysis of polyubiquitination of GFP-SOCS1 in HEK293T cells cotransfected with GFP-SOCS1, HA-Ub-K48 (R48K), and Myc-DDX4 as indicated and then treated with MG132 (10 µM, 4 h). (**F**) Immunoprecipitation analysis of polyubiquitination of GFP-SOCS1 in HEK293T cells cotransfected with GFP-SOCS1, HA-Ub-K63 (R63K), and Myc-DDX4 as indicated and then treated with MG132 (10 µM, 4 h). (**G**) Immunoprecipitation analysis of polyubiquitination of GFP-SOCS1-WT and GFP-SOCS1-K119R in HEK293T cells cotransfected with Myc-DDX4, GFP-SOCS1-WT, or GFP-SOCS1-K119R as indicated and then treated with MG132 (10 µM, 4 h). (**H**) Immunoprecipitation analysis of polyubiquitination of GFP-SOCS1 in HEK293T cells cotransfected with GFP-SOCS1, Myc-DDX4, and shUSP7 as indicated and then treated with MG132 (10 µM, 4 h). Data are shown as mean  ±  SD of three biological replicates (**A–G**).

It is well known that SOCS1 serves as a negative regulator of IFN-induced JAK–STAT signaling. On the basis of the above findings, we speculated that DDX4 may increase SOCS1 ubiquitination levels to positively regulate IFN-I-mediated antiviral activity. Our data showed that knockout of DDX4 significantly reduced Myc-SOCS1 ubiquitination levels under the circumstance of detecting overexpressed HA-Ub (Fig. 5A) or endogenous Ub (Fig. 5B). In turn, overexpression of DDX4 upregulated ubiquitination levels of GFP-SOCS1 (Fig. 5C and D), suggesting that DDX4 could mediate deubiquitinating cellular SOCS1. Polyubiquitination of protein substrates is commonly in the form of either K48- or K63-linked Ub chains. Given the deubiquitination of SOCS1 mediated by DDX4, we tried to determine whether DDX4 can affect the K48- and/or K63-linked ubiquitination of SOCS1. To this end, the types of SOCS1 ubiquitination were analyzed using two constructs, HA-Ub-R48K and HA-Ub-R63K, that retain only one lysine at position 48 (R48K) and position 63 (R63K), respectively. Our data showed that overexpression of DDX4 increased both K48- and K63-linked polyubiquitination chains of SOCS1 (Fig. 5E and F). What’s interesting is that the SOCS1 protein only has one lysine residue: K119. We mutated the K119 site to an inactivated R (K119R) and observed the effect of DDX4 overexpression on the ubiquitination level of Myc-SOCS1-K119R. The result showed that DDX4 increases the ubiquitination level of Myc-SOCS1-WT, but the K119R mutant is almost not ubiquitinated (Fig. 5G). Furthermore, we determined whether DDX4 directly ubiquitinated cellular SOCS1 and investigated the role of USP7 in the ubiquitination of SOCS1 mediated by DDX4. The results showed that DDX4 cannot obviously promote polyubiquitination of SOCS1 in USP7-deficient cells (Fig. 5H), indicating that DDX4 increased SOCS1 ubiquitination levels in a USP7-dependent manner. All in all, DDX4 elevates SOCS1 ubiquitination levels in a USP7-dependent manner.

### The negative regulation of SOCS1 protein levels and stability mediated by DDX4 in a USP7-dependent manner

**Fig 6:**
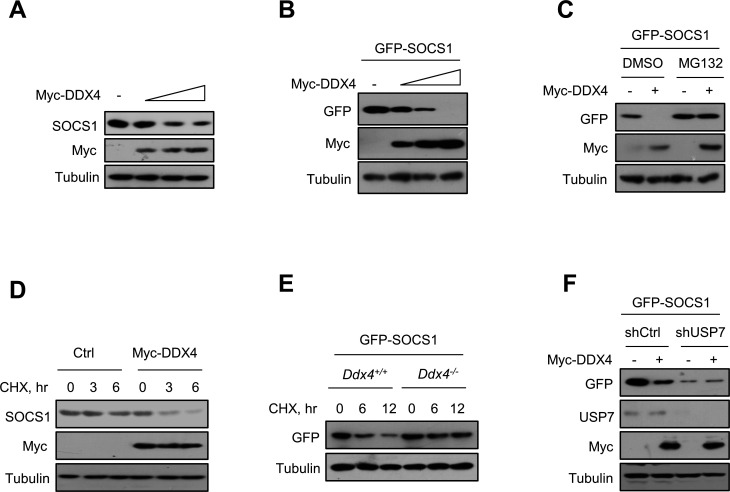
DDX4 negatively regulated the protein levels and stability of SOCS1 in a USP7-dependent manner. (**A**) Western blot analysis of SOCS1 levels in HEK293T cells transfected with an increasing amount of Myc-DDX4. (**B**) Western blot analysis of GFP-SOCS1 levels in HEK293T cells cotransfected with GFP-SOCS1 and an increasing amount of Myc-DDX4. (**C**) Western blot analysis of GFP-SOCS1 protein levels in HEK293T cells transfected with GFP-SOCS1, together with or without Myc-DDX4, followed by treatment with DMSO or MG132 (10 µM) for 12 h. (**D**) Western blot analysis of SOCS1 protein levels in RAW264.7 cells transfected with or without Myc-DDX4, followed by the treatment with CHX (50 mg/mL) as indicated. (**E**) Western blot analysis of GFP-SOCS1 protein levels in *Ddx4*^+/+^ or *Ddx4*^-/-^ RAW264.7 cells transfected with GFP-SOCS1, followed by the treatment with CHX (50 mg/mL) as indicated. (**F**) Western blot analysis of GFP-SOCS1 protein levels in HEK293T cells cotransfected with GFP-SOCS1, shCON, shUSP7, and Myc-DDX4 as indicated. Data are shown as mean  ±  SD of three biological replicates (**A–F**).

Given that DDX4 enhanced SOCS1 ubiquitination levels in a USP7-dependent manner, we questioned whether DDX4 negatively regulated SOCS1 protein levels. Our data showed that endogenous or exogenous SOCS1 presented a decreasing tendency along with increasing DDX4 (Fig. 6A and B). Furthermore, we asked whether the effect of DDX4 on SOCS1 protein levels is mediated by the Ub/proteasome system. We observed that the proteasome inhibitor MG132 was able to block the degradation of SOCS1 induced by DDX4 overexpression (Fig. 6C). Next, we wondered whether DDX4 could regulate SOCS1 protein stability. As such, a cycloheximide (CHX) pulse-chase assay was performed. CHX has been widely used for the inhibition of protein synthesis. We found that overexpression of DDX4 remarkably promoted the degradation of endogenous SOCS1 protein (Fig. 6D). But the degradation rate of SOCS1 was markedly decelerated when endogenous DDX4 was knocked out (Fig. 6E), indicating that SOCS1 stability is regulated by cellular DDX4. In addition, the knockdown of USP7 did not obviously decelerate the degradation rate of SOCS1 (Fig. 6F). Taken together, we demonstrate that DDX4 negatively regulated SOCS1 protein levels and stability in a USP7-dependent manner.

### The antiviral function of IFN-I regulated by DDX4 in a SOCS1/USP7-dependent manner

**Fig 7:**
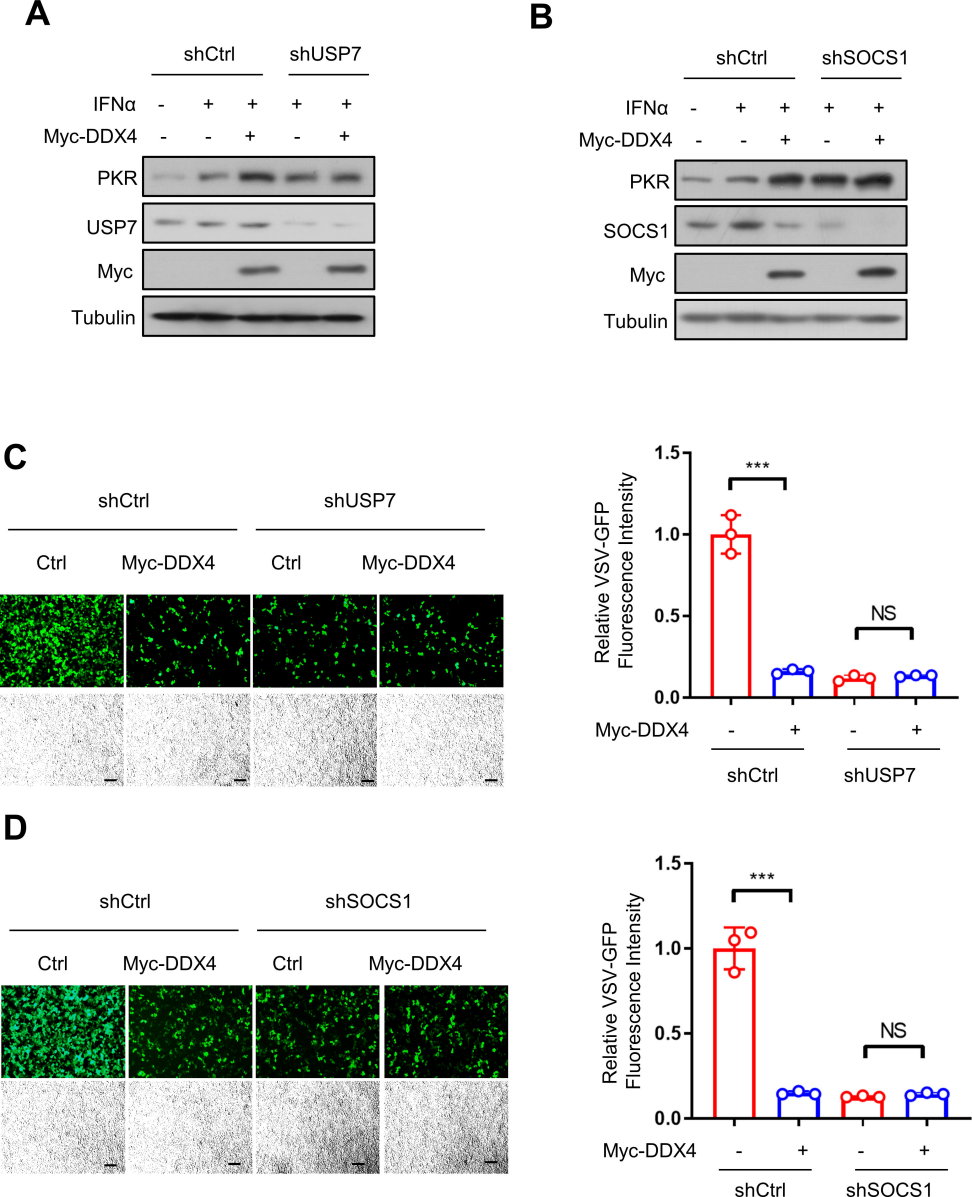
DDX4 regulated the antiviral function of IFN in a SOCS1/USP7-dependent manner. (**A**) Western blot analysis of PKR protein levels in HEK293T cells transfected with shCON or shUSP7, together with or without Myc-DDX4, followed by the treatment with IFNα (1,000 IU/mL) for 24 h. (**B**) Western blot analysis of PKR protein levels in HEK293T cells transfected with shCON or shSOCS1, together with or without Myc-DDX4, followed by the treatment with IFNα (1,000 IU/mL) for 24 h. (**C**) Fluorescence microscopy of VSV-GFP viruses and VSV-GFP fluorescence intensity in HEK293T cells transfected with shCON or shUSP7, together with or without Myc-DDX4. Scale bars, 100 µm. (**D**) Fluorescence microscopy of VSV-GFP viruses and VSV-GFP fluorescence intensity in HEK293T cells transfected with shCON or shSOCS1, together with or without Myc-DDX4. Scale bars, 100 µm. NS, not significant; ***P < 0.001. Data are shown as mean  ±  SD of three biological replicates (**A and B**).

We further elucidated whether the effect of DDX4 on the IFN-I antiviral response is dependent on SOCS1/USP7. As such, cells were transfected with shUSP7 or shSOCS1, together with or without Myc-DDX4, followed by treatment with IFNα. Our data demonstrated that the addition of DDX4 did not further increase PKR protein levels when either USP7 or SOCS1 was knocked down (Fig. 7A and B). Additionally, cells were transfected with shUSP7 or shSOCS1, together with or without Myc-DDX4, and then were infected with VSV-GFP. As expected, DDX4 did not decrease VSV-GFP infection in the knockdown of either USP7 or SOCS1 as shown by the GFP signal (Fig. 7C and D).

**Fig 8:**
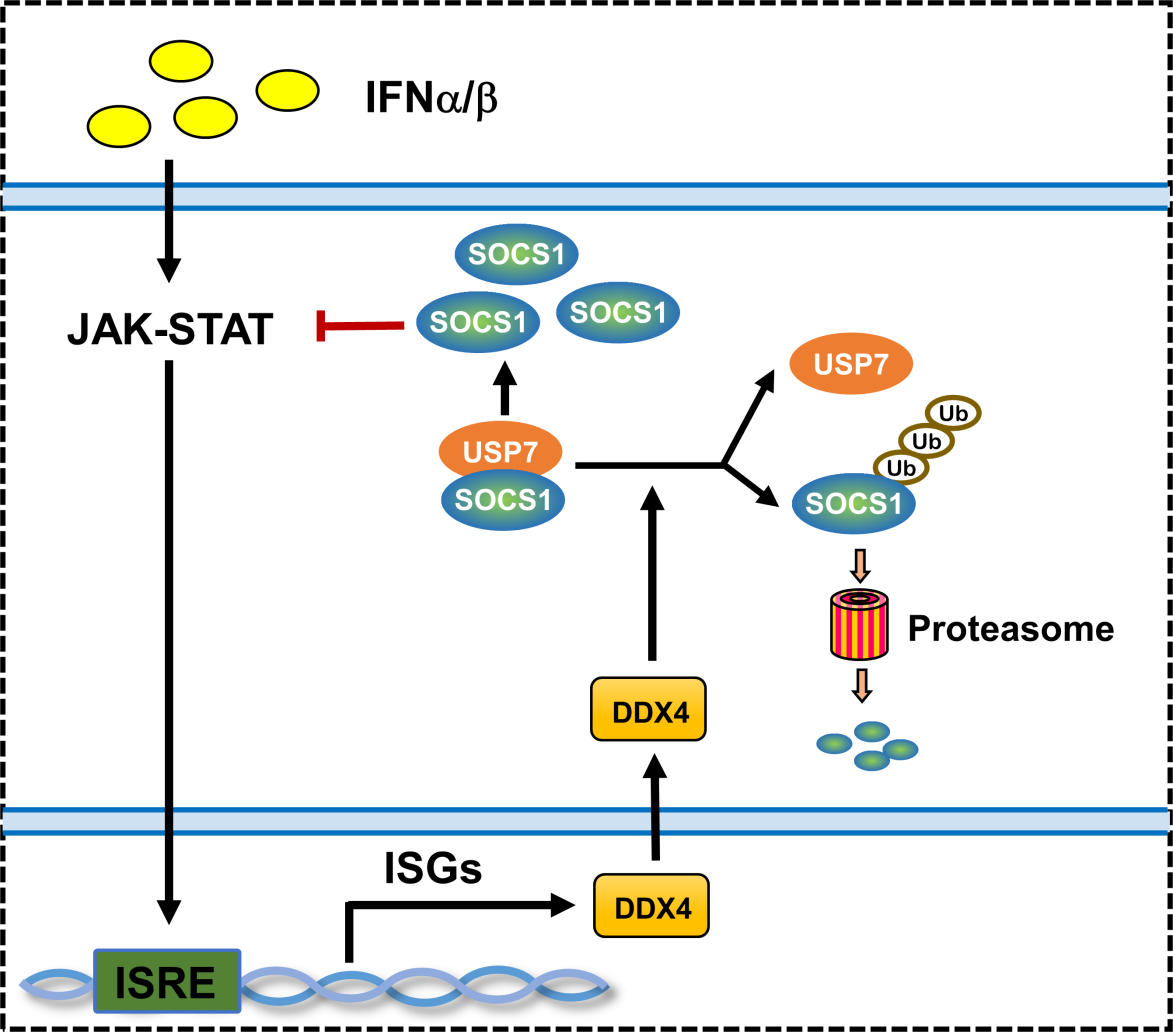
A proposed model of regulation of the IFN-JAK/STAT1 signaling by DDX4. (**A**) USP7 physically interacts with SOCS1 and enhances SOCS1 protein stability by deubiquitination effects, which in turn restricts the IFN-I-induced activation of JAK–signal transducer. DDX4 promotes host antiviral capacity by inhibiting the interaction between USP7 and SOCS1, which subsequently promotes the degradation of SOCS1.

Our study showed the novel role of DDX4 in regulating IFN-I response. Mechanistically, USP7 physically interacts with the SOCS1 and enhances SOCS1 protein stability by deubiquitination effects, which in turn restricts the IFN-I-induced activation of JAK–signal transducer. DDX4 promotes host antiviral capacity by inhibiting the interaction between USP7 and SOCS1, which subsequently enables the degradation of SOCS1 (Fig. 8). Taken together, we revealed that DDX4 regulated antiviral function of IFN-I in a SOCS1/USP7-dependent manner.

## DISCUSSION

DDXs act as multifunctional molecules that play an important biological role, with not completely characterized functions yet. Growing evidence demonstrates some DDX family members get involved in innate immunity, as is attested by the positive or negative regulatory role of DDX3, DDX5, DDX19, DDX24, DDX58, and DDX60 in mediating antiviral signaling pathways (10, 16–19). However, we know little about the physiological roles played by DDX4 in antiviral innate immunity. Previous studies mainly focused on the regulatory function of DDX4 in germline, stem cells, or tumor cells (11, 12). In this study, we identified a novel biological function of DDX4, unveiling that DDX4 serves as a positive regulatory molecule of IFN-I-mediated antiviral activity.

There is an interesting observation that viral infection or IFN-I increases DDX4 protein expression; in return, DDX4 promotes a cellular antiviral response by enhancing the IFN-mediated signaling pathway, forming a positive feedback loop to amplify the antiviral response. As such, disruption of this loop suggests a new mechanism for virus evasion.

To further uncover the potential mechanism of DDX4 in regulating IFN-I signaling, we attempted to use the predictive website to find the proteins interacting with DDX4 and observed a possible interaction of DDX4 with USP7. Interestingly, our previous study has demonstrated that USP7 can enhance SOCS1 protein stability by interacting with SOCS1 and deubiquitinating it, thus inhibiting IFN-I antiviral activity (4). Coincidentally, our previous findings and the predictive website pointed to the same interactive molecule, i.e., USP7. The present study took this as a breakthrough point to further explore the regulatory mechanism of IFN-I antiviral signaling mediated by DDX4. Then, we manifested that DDX4 interacted with USP7 but did not affect its protein levels, suggesting DDX4 might inhibit the interaction of USP7 with SOCS1. Afterward, DDX4 was proved to promote the degradation and stability of SOCS1 protein by increasing SOCS1 ubiquitination levels. As such, our findings revealed a positive regulatory mechanism of IFN-I antiviral activity mediated by the DDX4/USP7/SOCS1 axis.

Importantly, this negative regulation of SOCS1 mediated by DDX4 was dependent on the existence of USP7. IFN-I response is vital to induce host antiviral immunity. Undoubtedly, IFN-I-mediated signaling pathways are positively or negatively modulated by complex molecular regulatory networks. Our work demonstrated that the Ub-proteasome system plays an indispensable role in IFN-I regulation.

In summary, we have demonstrated that DDX4 can be upregulated by IFN-I and serves as a positive regulator of IFN-I-mediated antiviral activity. Mechanistically, DDX4 targets and binds with USP7, thus disrupting the interaction of USP7 and SOCS1 and promoting the degradation of SOCS1. Our study expands the understanding of DDX4 in the increase of IFN-I-based antiviral activity.

## MATERIALS AND METHODS

### Cell culture, maintenance, and transfection

RAW264.7, MEF, and human embryo kidney 293T cells were obtained from the American Type Culture Collection, with 2fTGH cells donated by Dr. Serge Y. Fuchs (University of Pennsylvania). In a humidified CO2 incubator (37°C in an atmosphere of 5% CO2), all cells were cultured in Dulbecco’s modified Eagle’s medium (DMEM; HyClone) supplemented with 10% fetal bovine serum (FBS; GIBCO, Life Technologies), 100 units/mL penicillin, and 100 µg/mL streptomycin. All transient transfections were conducted using either LongTrans (Ucallm), Lipofectamine 2000 (Thermo Fisher), or PEI (Polyetherimide) in accordance with the manufacturer’s instructions.

### Expression constructs and reagents

Myc-DDX4 was purchased from the YouBio company. Flag-USP7 was a gift from Dr. J. Wade Harper (Harvard Medical School, Addgene Plasmids). HA-USP7 was made using PCR amplification from Flag-USP7 and cloned into the HA-pcDNA3.1 vector. Myc-SOCS1 and GFP-SOCS1 were gifts from Dr. Serge Y. Fuchs (University of Pennsylvania). HA-Ub, HA-R48K, and HA-R63K were gifts from Dr. Lingqiang Zhang (State Key Laboratory of Proteomics, Beijing). All shRNAs against USP7 (shUSP7) or STAT1 (shSTAT1) were purchased from GENECHEM (Shanghai, China). DNA sequencing was used to verify all plasmids. Recombinant human IFNα was purchased from PEPROTECH, and recombinant mouse IFNβ (mIFNβ) was purchased from the R&D Systems. Additionally, MG132, CHX, polybrene, and puromycin were purchased from Sigma-Aldrich.

### Viral infection *in vitro*

VSV and SeV were gifts from Dr. Chen Wang (China Pharmaceutical University), with HSV type 1 from Chunfu Zheng (Fujian Medical University) and H1N1 (PR/8/34) from Dr. Jianfeng Dai (Soochow University, China). Cells were washed twice with 1 × PBS and cultured in serum-free medium, followed by being infected with viruses serially diluted with DMEM for 1.5 h. After supernatant removal, the cells were cultured in DMEM medium with 10% FBS until harvest.

### TCID_50_ assay

VSV viral titers were determined by a standard 50% tissue culture-infective dose (TCID_50_) assay. In brief, following the collection of the cell culture supernatants containing viruses and serial dilution with DMEM, diluted supernatants were applied to 96-well plates containing Vero cells monolayers. The TCID_50_ was calculated using the Spearman–Karber algorithm.

### CHX pulse-chase assay

Using CHX pulse-chase assays, the half-life of SOCS1 protein was measured. Generally, cells were transfected with or without Myc-DDX4 plasmids. After 48 h, cells were treated with DMSO or CHX (50 µg/mL) as indicated. Following cell harvest, whole cell lysates were boiled and analyzed by western blot.

### *In vivo* ubiquitination assay

The corresponding plasmids, along with or without HA-Ub, were transfected into the cells. Following transfection for 48 or 72 h, cells were harvested in RIPA (Radio Immunoprecipitation Assay) strong lysis buffer with Nethylmaleimide (10 mM), PMSF (phenylmethanesulfonyl fluoride) (50 µg/mL), and protease inhibitor mixtures (Sigma-Aldrich).

Immunoprecipitation was conducted using a specific antibody (Ab) on a rotor at 4°C for analysis of SOCS1 ubiquitination. Western blot analysis was performed using the anti-HA or anti-Ub Ab after the immunoprecipitates were washed three times in high-salt (500 mM NaCl) washing buffer and twice in normal salt (150 mM NaCl) washing buffer.

### Fluorescence microscopy

Cells infected with VSV-GFP were imaged under an upright fluorescence microscope at a magnification of ×200. Data were analyzed by FlowJo software (FlowJo, Ashland, OR).

### Total RNA extraction

With TRIzol reagent (Invitrogen), RNA was extracted from different types of cells. As per the manufacturer’s instructions, cDNA was produced from 1 µg of total RNA by reverse transcription using oligo (dT) or random primers.

### Real-time quantitative PCR

RT-qPCR was performed with the SYBR Green (Selleck) using a StepOne Plus real-time PCR system (Applied Bioscience). The relative gene expression levels were calculated based on the change-in-cycling-threshold (2^-ΔΔCt^) method. Quantification of all target genes was normalized to the control gene β-actin, and all data are shown as fold change normalized to that in either unstimulated or uninfected cells accordingly. The results were presented as the average mean ± SD of three independent experiments. The primer sequences are as following:

Human *DDX4:*

Forward: 5′-GTGTCTGGACATGATGCACCAC-3′

Reverse: 5′-GCAAGCCATCAAATCTCGTCCTG-3′

Mouse *Ddx4:*

Forward: 5'- GGACGAGATTTGATGGCTTGTGC-3′

Reverse: 5'- AGCGACTGGCAGTTATTCCATCC-3′

VSV:

Forward: 5′-ACGGCGTACTTCCAGATGG-3′

Reverse: 5′-CTCGGTTCAAGATCCAGGT-3′

SeV:

Forward: 5′-GATGACGATGCCGCAGCAGTAG-3′

Reverse: 5′-CCTCCGATGTCAGTT GGTTCACTC-3′

H1N1:

Forward: 5′-TTCTAACCGAGGTCGAAACG-3′

Reverse: 5′-ACAAAGCGTCTACGCTGCAG-3′

HSV:

Forward: 5′-CCAACGCCAAGACGGTGTA-3′

Reverse: 5′-GGGGGTCGTGAGGAAGAAC-3′

Mouse *If*n*β*:

Forward: 5′-CTTCGTGTTTGGTAGTGATGGT-3′

Reverse: 5′-GGGGATGATTTCCAGCCGA-3′

Human *IFIT1*:

Forward: 5′-CACAAGCCATTTTCTTTGCT-3′

Reverse: 5′-ACTTGGCTGCATATCGAAAG-3′

β-actin

Forward: 5′-ACCAACTGGGACGACATGGAGAAA-3′

Reverse: 5′-ATAGCACAGCCTGGATAGCAACG-3′

### Western blot

Equal amounts of total proteins were subjected to SDS-PAGE and then transferred to PVDF (polyvinylidene fluoride) membranes (Millipore). Membranes were blocked in either 5% skim milk or 5% BSA (albumin from bovine serum) for 0.5 h at room temperature and probed with the corresponding primary antibodies, followed by the respective HRP (Horseradish Peroxidase)-conjugated goat anti-mouse or anti-rabbit (Bioworld) secondary Ab. The membrane was visualized with the ECL Prime (Thermo Scientific) after being washed three times with PBST (Phosphate Buffered Saline with Tween 20). The antibodies with indicated dilutions were as follows: DDX4 (Affinity, AF4098, 1:500), VSV-G (Santa Cruz, sc-66180, 1:2,000), PKR (Cell Signaling Technology, sc-6282, 1:1,000), IFIT1 (Santa Cruz, sc-134948, 1:1,000), GFP (Santa Cruz, sc-9996, 1:3,000), p-STAT1 (Cell Signaling Technology, 9617, 1:1,000), STAT1 (Cell Signaling Technology, 8826, 1:5,000), HA (Sigma, ab9110, 1:5,000), Myc (Abmart, M20002, 1:5,000), Ub (Santa Cruz, sc-8017, 1:500), USP7 (Santa Cruz Biotechnology, sc-30164, 1:1000), SOCS1 (Millipore, Billierica, MA; 04–002, 1:1,000), IFNAR1 (Abcom, ab45172, 1:1,000), and Tubulin (Proteintech, 66031–1-Ig, 1:5,000).

### Immunoprecipitation

Cells were harvested using the lysis buffer containing 150 mM NaCl, 1% Nonidet P-40, 0.5 mM EDTA, 20 mM Tris-HCl (pH 7.4), PMSF (50 µg/mL), and protease inhibitors mixtures (Sigma). N-ethylmaleimide (10 mM) was added into RIPA buffer containing SDS (Beyotime) to examine protein ubiquitination. At 4°C, the cell lysates were incubated with speciﬁc Abs on a rotor, followed by the addition of twice-washed Protein G-agarose beads (Millipore; 16–266) into the supernatant. Then, the mixture was incubated for 2–3 h on a rotor at 4°C. For immunoprecipitation of Flag/Myc/HA-tagged proteins, M2 afﬁnity gel (A2220; Sigma‒Aldrich) and Myc or HA magnetic beads (Selleck) were added to cell lysates. Following three washes with the washing buffer containing 150 mM NaCl, the immunoprecipitates were analyzed by western blot. For IP (Immunoprecipitation) normalization, target proteins were first immunoprecipitated and then were serially diluted with a loading buffer for analysis of target protein levels by immunoblotting. In accordance with the immunoblotting results, the same amount of immunoprecipitated target proteins was loaded for analysis of interaction or ubiquitination. The whole cell lysates (30 µg) were used for an input control.

### CRISPR-Cas9-mediated genome editing

HEK293T cells were transfected with small guide RNA targeting human DDX4 (5′-ATTGGGCGTACTGGTCGTTG-3′), which was cloned into the lentiCRISPRv2 vector. After 48 h of transfection, the supernatant was used to infect RAW264.7 cells. Then the RAW264.7 cells were cultured under puromycin selection until further experiments.

### *In vitro* binding assay

Cells were transfected with either Flag-USP7 or Myc-DDX4 for 48 h. Flag M2 beads were used to pull down Flag-USP7, and Myc Ab and Protein-A/G beads were used to pull down Myc-DDX4. After washing three times, Flag-USP7 immunoprecipitates were eluted by Flag peptides (Sigma, Cat #F3290), and Myc-DDX4 immunoprecipitates were eluted by Myc peptides (Beyotime, Cat #P9805). Recombinant human SOCS1 Fusion protein was purchased from Proteintech (Cat #Ag19318). Flag-USP7 and Myc-DDX4 were added to SOCS1 for binding reaction for 2 h at room temperature. Immunoprecipitation was performed in the reaction buffer by a specific SOCS1 Ab, and Flag-USP7 was analyzed by immunoblotting using Flag Ab.

### Statistical analysis

Statistical comparisons between groups were conducted using the two-tailed unpaired Student’s *t* test. Values of *P* < 0.05 were considered statistically signiﬁcant. *P < 0.05, **P < 0.01, and ***P < 0.001; NS, not significant.
